# Prognosis and therapy of tumor-related versus non-tumor-related status epilepticus: a systematic review and meta-analysis

**DOI:** 10.1186/1471-2377-14-152

**Published:** 2014-07-19

**Authors:** Yunus Arik, Frans SS Leijten, Tatjana Seute, Pierre A Robe, Tom J Snijders

**Affiliations:** 1 University Medical Center Utrecht, P.O. Box 85500, Utrecht 3508, GA, The Netherlands; 2 Brain Center Rudolf Magnus, Department of Neurology, University Medical Center Utrecht, P.O. Box 85500, Utrecht 3508, GA, The Netherlands; 3 Brain Center Rudolf Magnus, Department of Neurosurgery, University Medical Center Utrecht, P.O. Box 85500, Utrecht 3508, GA, The Netherlands

**Keywords:** Brain tumor, Meta-analysis, Neuro-oncology, Prognosis, Status epilepticus, Therapy

## Abstract

**Background:**

Status epilepticus (SE) is a medical emergency with high mortality rates. Of all SE’s, 7% are caused by a brain tumor. Clinical guidelines on the management of SE do not make a distinction between tumor-related SE and SE due to other causes. However, pathophysiological research points towards specific mechanisms of epilepsy in brain tumors. We investigated whether clinical features support a distinct profile of tumor-related SE by looking at measures of severity and response to treatment.

**Methods:**

Systematic review of the literature and meta-analysis of studies on adult SE that report separate data for tumor-related SE and non-tumor-related SE on the following outcomes: short-term mortality, long-term morbidity, duration of SE, and efficacy of anticonvulsant intervention.

**Results:**

Fourteen studies on outcome of SE were included. Tumor-related SE was associated with higher mortality than non-tumor-related SE (17.2% versus 11.2%, RR 1.53, 95%-CI 1.24-1.90). After exclusion of patients with hypoxic-ischemic encephalopathy (a group with a known poor prognosis) from the non-tumor-group, the difference in mortality increased (17.2% versus 6.6%; RR 2.78, 95%-CI 2.21 – 3.47). Regarding long-term morbidity and duration of SE there were insufficient data. We did not find studies that systematically compared effects of therapy for SE between tumor- and non-tumor-related SE.

**Conclusions:**

Based on – mostly retrospective – available studies, short-term mortality seems higher in tumor-related SE than in SE due to other causes. Further studies on the outcome and efficacy of different therapeutic regimens in tumor-related SE are needed, to clarify whether tumor-related SE should be regarded as a distinct clinical entity.

## Background

Epilepsy is very common in patients with brain tumors; depending on the type of tumor, estimates of the frequency of epilepsy vary between 10 and 100% [1]. While 30 to 50% of the patients with a brain tumor present with seizures, 10 to 30% develop seizures in the course of the disease [2-5]. In low-grade tumors, slow growth probably gives rise to the development of focal or remote cell changes. These cell changes are associated with epileptogenesis. Rapidly progressive tumors (high-grade lesions) are thought to induce epilepsy through abrupt tissue damage [1].

Continuous seizure activity of five minutes poses an increased risk of status epilepticus (SE) [6]. Seven percent of all adult SE are caused by a brain tumor [7]. In most national and international guidelines, SE is considered a single clinical entity, although there is a consensus that partial and non-convulsive forms of SE should not be treated as aggressively as generalized convulsive SE. In these types of SE, the risk of intensive sedative treatment and intubation probably does not outweigh the benefits of seizure control [8]. Most clinical guidelines on the management of SE do not make a distinction between tumor-related SE and SE due to other causes.

However, recent pathophysiological studies point towards specific mechanisms of epilepsy in brain tumors. The tumor is, through invasion and infiltration, responsible for alterations in receptor expression and neurotransmitters, leading to changes in the peritumoral tissue. These changes contribute to the imbalance of excitation and inhibition in brain networks, leading to epileptogenesis. The alterations in neurotransmitters and receptor expression may provide targets for drug therapy (for a review, see ref. [9]). The finding of overexpression of multi-drug transporters in patients with brain tumors further suggests that tumor-related epilepsy and SE require a specific therapeutic strategy [9]. Until now, no particular anticonvulsant has been shown to be superior to others in treating adults with brain tumor-related epilepsy [10].

These pathophysiological and pharmacological data point towards a specific mechanism underlying tumor-related SE, and suggest that the efficacy of available anticonvulsants may differ between tumor-related SE and SE due to other causes. However, in the current clinical situation, SE is now treated uniformly regardless of underlying cause. If tumor-related SE is truly characterized by a specific underlying mechanism and sensitivity to anticonvulsants, this might be reflected in a different clinical outcome between tumor-related SE and SE due to other causes.

To study potential differences between tumor-related SE and SE due to other causes, we conducted a systematic review of the literature and a meta-analysis. We aimed to answer the following questions:

1. Does outcome in tumor-related SE differ from SE due to other causes in terms of short-term mortality, long-term morbidity (neurological deficits lasting beyond the initial post-ictal period) and duration of SE?

2. Does the efficacy of particular treatments differ between tumor-related SE and SE due to other causes?

## Methods

We performed two systematic searches in the PubMed database. The results have been updated until January 12, 2013. No ethical committee approval was deemed necessary for this literature review and meta-analysis.

### Outcome of status epilepticus

For the first search, the term *status epilepticus* was combined with *outcome, mortality, morbidity, fatality, prognosis, coma, death, incidence, prevalence* and *epidemiology.* For a paper to be included in this literature review, status epilepticus had to be defined as a single continuous seizure or a series of epileptic seizures with clouded consciousness between ictal events; although most studies used a minimum duration of 30 minutes in the definition (in accordance with traditional guidelines), we did not use this as an inclusion criterion since modern data and guidelines use less stringent time criteria [6]. We limited the search to human studies on adults written in English, Dutch or Turkish. We excluded review articles, case reports and treatment protocols. Full text screening and reviewing of the residual studies was conducted. We included studies on generalized and focal SE (or both), but excluded studies that focused on specific subgroups of SE such as refractory SE, elderly patients only or critically ill patients. To be included, a study had to have more than 5 tumor patients in the study group. Also, information on one or more of the following outcomes had to be available: short-term mortality, long-term morbidity and duration of SE. The screening and selection of papers from the original search was performed by one author (YA) and reviewed by the last author (TJS).

We defined mortality as short-term mortality, and we included data on 30-day mortality, case fatality or mortality at discharge. Long-term morbidity was defined as the occurrence of new neurological deficits that lasted beyond the regular post-ictal period. To be included, data had to be available on (a) either the ‘back to baseline’-percentages, or – conversely – the percentages of patients who had worsened clinically after SE, and (b) mortality. For inclusion in the analysis on duration of SE, data had to be available on the mean SE duration (in minutes or hours). For all the outcomes, we extracted separate data for tumor-related SE versus SE due to other causes.

Within the subgroup of SE due to causes other than tumors, the subgroup of patients with hypoxic/anoxic encephalopathy (HAE) after cardiopulmonary resuscitation represents a specific subgroup, in which the occurrence of SE itself is associated with a very poor prognosis [11]. In these cases, the mortality is thought to be the consequence of the disease itself (HAE) rather than of the SE. To exclude an effect of such HAE-associated mortality on the outcomes, the outcome analyses were repeated after exclusion of cases with HAE-associated SE.

Authors were contacted by email if a study had missing or incomplete information.

Based on the data from the separate studies, we calculated the total number of patients with tumor-related SE and patients with SE due to another cause. We then calculated total mortality and morbidity and the median duration of SE for both groups. From these grouped data, we calculated the weighted averages for all outcomes, and expressed the difference between patient groups as relative risk (RR) and 95% confidence interval (95%-CI) for mortality and morbidity. For comparison of SE duration between groups, we used the Mann-Whitney test.

### Anticonvulsant therapy

An extensive search query was applied to find studies on the efficacy of anticonvulsant therapy in treating tumor-related SE, rather than SE treatment in general. The search query can be found in the Additional file 1. We applied the same limits as in the search on outcome. Treatment protocols and reviews were excluded. This method resulted in a very small number of studies. Therefore, we chose to include all studies with more than one patient with tumor-related SE. The data presentation is descriptive in nature, since no formal quantitative analysis (meta-analysis) could be performed.

## Results

**Figure 1:**
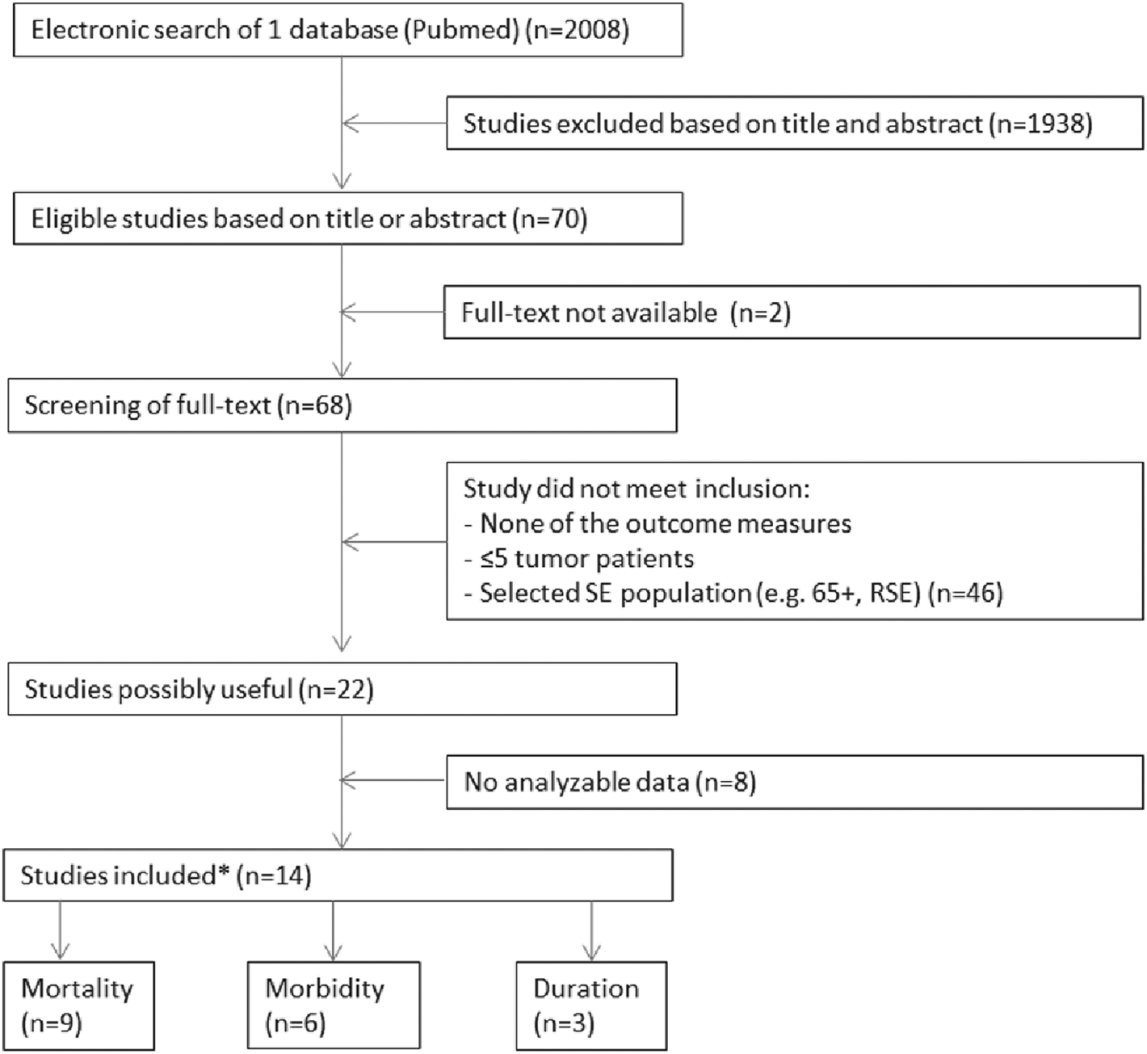
**Flowchart of the search for the prognosis of tumor-related status epilepticus versus status epilepticus by other causes.** *Some studies had data for 2 outcomes. SE = status epilepticus, RSE = refractory status epilepticus.

The results of the search concerning outcome of SE are given in Figure 1. Of the fourteen studies that met the criteria, important data on the outcome measures were missing in nine studies. We tried to obtain these non-reported data through the corresponding author. If this did not result in additional data we categorized the study as ‘no analyzable data’, which was the case in three studies that met the initial inclusion criteria for the outcome mortality, leaving nine analyzable studies. Of these nine studies with valid mortality data, one study did not provide separate data on cases of HAE; consequently, eight studies were included in the analysis with exclusion of HAE cases.

For the analysis on long-term morbidity, we did not leave out any study. For the analysis on SE duration, six studies only mentioned the median duration or range, in which case we had to categorize them as ‘no analyzable data’.

### Short-term mortality

The data from the nine studies that we included can be found in Table 1. Short-term mortality in tumor-related SE was higher than in SE due to other causes (weighted average 17.2% versus 11.3%, RR 1.53, 95%-CI 1.24-1.90). After exclusion of cases of HAE, this difference increased (tumor-related SE 17.2% versus non-tumor-related SE 6.5%; RR 2.66, 95%-CI 2.14 – 3.31).

**Table 1:** Mortality in tumor-related status epilepticus versus status epilepticus due to another cause

***Author***	***Total no of patients***	***No of tumor patients***	***Mortality in tumor group***	***Mortality in non-tumor group***	***Mortality in non-tumor group, HAE excluded***	***Type of study***	***Setting***
Amare [12]	119	13	23.1%	19.8%	18.4%	Retrospective	Tertiary hospital
Cavaliere [13]^*^	35	34	23.0%	0%	0%	Retrospective	University hospital
Chen [14]^**#^	220	17	11.8%	15.3%	n/a^#^	Prospective	University hospital
DeLorenzo [7]	137	10	30.0%	36.2%	31.4%	Prospective	Population based
Li [15]	203	15	13.3%	16.0%	16.1%	Retrospective	University hospital
Rossetti [16]	96	14	21.4%	14.6%	14.6%	Prospective	University hospital
Scholtes [17]	236	12	16.7%	14.7%	14.8%	Retrospective	Hospitals and epilepsy centers
Towne [18]	253	11	36.0%	22.7%	17.5%	Retrospective	Hospital
Wu [19]	15601	291	15.5%	10.7%	5.8%	Retrospective	Population based
***Total***	**16900**	**417**					
***Weighted averages***			**17.2**%	**11.3**%	**6.5**%		

*This study only included tumor patients; one patient had a systemic tumor without brain-metastasis. ^**^Brain neoplasm and developmental malformation were in the same group. Separate data could not be obtained. ^#^No separate data on hypoxic/anoxic encephalopathy were given. HAE = hypoxic/anoxic encephalopathy.

### Long-term morbidity

Only one study (*Scholtes et al*.) [17] made a distinction between morbidity for tumor-related SE and other causes of SE. All other studies only mentioned the morbidity in the total group. In the study by *Scholtes et al.,* 33.3% of the patients in the tumor group were clinically worse than before the SE, 50% recovered and 16.7% died. For the group of patients with SE from other causes, the numbers were 15.2%, 70.1% and 14.7% respectively (RR for long-term morbidity or death versus complete recovery 1.67, 95%-CI 0.92-3.05).

### Duration

We found three studies with data on SE duration, of which only one study specified the duration for tumor-related SE [20]. Mean duration of the SE in the tumor group was 152.9 (standard error 43.0, median 70.0) minutes versus 174.1 (standard error 18.6, median 85.0) minutes in SE due to other causes.

### Anticonvulsant therapy

The search on anticonvulsant therapy in patients with tumor-related SE resulted in three articles with more than one tumor-related SE (see Additional file 2). Given this paucity of data and the lack of prospective comparative series, it was not possible to formally compare data between tumor-related and non-tumor-related SE. The available descriptive data can be found in Additional file 3.

## Discussion

In this systematic search and meta-analysis on differences in outcome between status epilepticus (SE) due to a brain tumor and SE due to other causes, we found a significantly higher mortality for tumor-related SE (17.3% versus 11.2%, RR 1.53, 95%-CI 1.24-1.90). This excess mortality in tumor-related SE was even more pronounced after exclusion of patients with hypoxic-ischemic encephalopathy from the non-tumor group (17.2% versus 6.5%, RR 2.66, 95%-CI 2.14 – 3.31). Available data on long-term morbidity and duration of SE were insufficient to draw any definite conclusions. Regarding long-term morbidity, the study by *Scholtes et al.*[17] shows a non-significant increase in morbidity in tumor-related SE (33.3% versus 15.2% in SE due to other causes). As for the duration of the SE, *Legriel et al.*[20] found a shorter duration in tumor-related SE (152.9 versus 174.1 minutes).

There is a substantial heterogeneity in mortality data, which increases the risk of bias; of note, the large-scale study by *Wu et al.*[19] largely determined our findings on mortality, since it provides 92% of the patients in this analysis. Another limitation of our analysis was that most of the studies initially included were mainly about SE in general (with the study by *Cavaliere et al*. [13] as an exception), and separate data for tumor patients and non-tumor patients were not available; for most of the studies, these specific data could no longer be retrieved from the authors. These unobtainable data limited comparative analysis for the outcome measures long-term morbidity and SE duration.

The reviewed studies do not provide data on the role of different types of brain tumors. This limits the interpretation of the data, since previous literature has shown that different brain tumors greatly differ in epileptogenicity [1]. Also, the preclinical and translational data that point towards a distinct pathophysiological mechanism of epilepsy and SE in patients with brain tumors, were mostly derived from patients with gangliogliomas and other glial tumors [9]. Future studies on tumor-related SE should report separately for the different types of brain tumors, in order to elucidate whether our findings on tumor-related SE apply for all brain tumors, or whether they are specific to certain tumor types. In addition, further studies should investigate whether the occurrence and outcome of tumor-related SE is dependent on (previous or current) anti-tumor-treatment such as radio- and chemotherapy, since several anti-tumor treatments are associated with reduction of epilepsy burden [1].

It is uncertain whether the increased mortality in tumor-related SE is the consequence of the SE itself (and its treatment). Progression of the underlying brain tumor or the possibility of selection of certain – prognostically unfavorable – subgroups in the included studies may (also) explain excess mortality.

The few articles on the efficacy of anticonvulsant(s) in treating tumor-related SE versus SE due to other causes were too heterogeneous and of insufficient quality to permit any formal analysis. This finding supports the notion that tumor-related SE is not yet considered a separate clinical entity.

## Conclusion

Pooled data from observational, mostly retrospective, studies show that tumor-related status epilepticus is associated with a statistically significant increase in mortality compared to status epilepticus by other causes. Available low-quality data suggest a higher rate of long-term morbidity and shorter duration of status epilepticus in tumor-related status epilepticus than in status epilepticus by other causes. However, data interpretation is hindered by the heterogeneity between studies and the lack of data on tumor subtypes and possible underlying tumor progression.

Based on the available data, no conclusion can be formulated on the efficacy of particular anticonvulsants in tumor-related SE versus SE due to other causes.

Further research is needed to provide more data about mortality, long-term morbidity, duration of the status epilepticus, and efficacy of anticonvulsants in tumor-related status epilepticus. Future studies on outcome of SE should treat tumor-related SE as a separate entity.

## Abbreviations

95%-CI: 95% confidence interval; HAE: Hypoxic/anoxic encephalopathy; RR: Relative risk; RSE: Refractory status epilepticus; SE: Status epilepticus.

## Competing interests

The authors declare that they have no competing interests.

TJS is financially supported by the Ton & Patricia Bohnenn Fund for Neuro-Oncology Research. The authors performed all aspects of this study (design, data acquisition, analysis, writing of this paper, decision to submit) independently of this funding source.

## Authors’ contributions

All authors contributed to the conception of the study. The screening and selection of papers from the original search was performed by YA and reviewed by TJS. YA and TJS drafted the manuscript. FSSL, TS and PAR critically reviewed the manuscript and gave important input. All authors have approved the final version of the manuscript.

## Pre-publication history

The pre-publication history for this paper can be accessed here:

http://www.biomedcentral.com/1471-2377/14/152/prepub

## Supplementary Material

Additional file 1 Search query on Anticonvulsant therapy. Click here for file

Additional file 2 Flowchart of the search on anticonvulsant therapy in tumor-related status epilepticus. Click here for file

Additional file 3 Studies on therapy of status epilepticus. Click here for file
