# Supplementary material for: Prognosis and therapy of tumor-related versus non-tumor-related status epilepticus: a systematic review and meta-analysis
Source: BMC Neurol. 2014 Jul 19;14:152. doi: 10.1186/1471-2377-14-152 (PMC4108966; doi:10.1186/1471-2377-14-152)
Supplement: Additional file 1 — Search query on Anticonvulsant therapy. [file 1471-2377-14-152-S1.docx]

**Additional file 1 (Appendix): Search query on Anticonvulsant therapy**

*Appendix to: Arik Y, Leijten FS, Seute T, Robe PA, Snijders TJ. Prognosis and therapy of tumor-related versus non-tumor-related status epilepticus: a systematic review and meta-analysis.*

#1 MeSH descriptor Brain Neoplasms explode all trees

#2 cancer* or tumor* or tumour* or malignan* or carcinoma* or neoplasm*

#3 brain

#4 astrocyt*

#5 oligodendrog*

#6 ependym*

#7 choroid plexus

#8 neuroepithel*

#9 neuronal or neuronal-glial

#10 pineal*

#11 embryonal

#12 haemopoietic or hemopoietic

#13 germ cell*

#14 meninge*

#15 sella*

#16 central nervous system or CNS

#17 metastasis

#18 (#3 AND #17)

#19 (#3 OR #4 OR #5 OR #6 OR #7 OR #8 OR #9 OR #10 OR #11 OR #12 OR #13 OR #14 OR #15 OR #16)

#20 (#2 AND #19)

#21 (#1 OR #18 OR #20)

#22 MeSH descriptor Status epilepticus explode all trees

#23 MeSH descriptor Epilepticus partialis continua explode all trees

#24 (#22 OR #23)

#25 MeSH descriptor Anticonvulsants explode all trees

#26 anticonvulsant*

#27 phenytoin

#28 valproic acid or sodium valproate

#29 phenobarbitone

#30 phenobarital

#31 oxcarbazepine

#32 carbamazepine

#33 lamotrigine

#34 gabapentin

#35 pregabalin

#36 vigabatrin

#37 levetiracetam

#38 tiagabine

#39 topiramate

#40 rufinamide

#41 lacosamide

#42 zonisamide

#43 clobazam

#44 midazolam

#45 ethosuximide

#46 (#25 OR #26 OR #27 OR #28 OR #29 OR #30 OR #31 OR #32 OR #33 OR #34 OR #35 OR #36 OR #37 OR #38 OR #39 OR #40 OR #41 OR #42 OR #43 OR #44 OR #45)

#47 (#21 AND #24 AND #46)
