# Supplementary material for: Prognosis and therapy of tumor-related versus non-tumor-related status epilepticus: a systematic review and meta-analysis
Source: BMC Neurol. 2014 Jul 19;14:152. doi: 10.1186/1471-2377-14-152 (PMC4108966; doi:10.1186/1471-2377-14-152)
Supplement: Additional file 3 — Studies on therapy of status epilepticus. [file 1471-2377-14-152-S3.docx]

Additional file 3

Online-only table S1: studies on therapy of status epilepticus.

*Supplement to: Arik Y, Leijten FS, Seute T, Robe PA, Snijders TJ. Prognosis and therapy of tumor-related versus non-tumor-related status epilepticus: a systematic review and meta-analysis.*

| Studies | Cranford[21] | Gamez-Levva[22] | Kang [23] | Mnatsakanyan[24] | Swisher[25] |
| --- | --- | --- | --- | --- | --- |
| **T**otal no of SE patients | 25 | 34 | 1 | 10 | 23 |
| **T**otal no of tumor-related SE | 1 | 4 | 1 | 2 | 23 |
| **I**ntervention | Phenytoin mean 16.4±2.7mg/kg | Levetiracetam 500-1500mg 2dd | Botulinum toxin | Lacosamide i.v. 100-200mg 2dd | median daily dose PGB 375mg, LEV 3000mg, serum PHT lvl 15-25microg/ml |
| **A**dditional information | - | Levetiracetam 500mg in 100ml saline, 1000mg median loading dose. | 200 Mouse units i.m. in AED resistant EPC. | 200-300mg i.v. median loading dose. | - |
| **S**eizure free in total group (%) | 77.8 | 44.1 | - | 70 | - |
| **S**eizure reduction in total group (%) | - | - | - | 10 | - |
| **N**o effect in total group (%) | - | 29 | - | 20 | - |
| **S**eizure free in tumor group (%) | - | - | - | 50 | 70 |
| **S**eizure reduction in tumor group (%) | - | - | 100 | 50 | 0 |
| **N**o effect in tumor group (%) | - | - | - | 0 | 30 |

PGB=pregabalin, LEV=levetiracetam, PHT=phenytoin, AED= antiepileptic drugs, EPC=epilepsia partialis continua.

**References belonging to Online-only table S1**

21. Cranford RE, Leppik IE, Patrick B, Anderson CB, Kostick B (1979) Intravenous phenytoin in acute treatment of seizures. Neurology 29:1474-1479.

22. Gamez-Leyva G, Aristin JL, Fernandez E, Pascual J (2009) Experience with intravenous levetiracetam in status epilepticus: a retrospective case series. CNS Drugs 23:983-987.

23. Kang JS, Krakow K, Roggendorf J, Steinmetz H, Hilker R (2009) Botulinum toxin treatment of epilepsia partialis continua. Mov Disord 24:141-143.

24. Mnatsakanyan L, Chung JM, Tsimerinov EI, Eliashiv DS (2012) Intravenous Lacosamide in refractory nonconvulsive status epilepticus. Seizure 21:198-201.

25. Swisher CB, Doreswamy M, Gingrich KJ, Vredenburgh JJ, Kolls BJ (2012) Phenytoin, levetiracetam, and pregabalin in the acute management of refractory status epilepticus in patients with brain tumors. Neurocrit Care 16:109-113.
